# How Evolving Heterogeneity Distributions of Resource Allocation Strategies Shape Mortality Patterns

**DOI:** 10.1371/journal.pcbi.1002825

**Published:** 2013-01-17

**Authors:** Yann Le Cunff, Annette Baudisch, Khashayar Pakdaman

**Affiliations:** 1 Institut Jacques Monod, CNRS UMR 7592, Univ Paris Diderot, Paris Cité Sorbonne, Paris, France; 2 Max Planck Institute for Demographic Research, Rostock, Germany; University of New South Wales, Australia

## Abstract

It is well established that individuals age differently. Yet the nature of these inter-individual differences is still largely unknown. For humans, two main hypotheses have been recently formulated: individuals may experience differences in aging rate or aging timing. This issue is central because it directly influences predictions for human lifespan and provides strong insights into the biological determinants of aging. In this article, we propose a model which lets population heterogeneity emerge from an evolutionary algorithm. We find that whether individuals differ in (i) aging rate or (ii) timing leads to different emerging population heterogeneity. Yet, in both cases, the same mortality patterns are observed at the population level. These patterns qualitatively reproduce those of yeasts, flies, worms and humans. Such findings, supported by an extensive parameter exploration, suggest that mortality patterns across species and their potential shapes belong to a limited and robust set of possible curves. In addition, we use our model to shed light on the notion of subpopulations, link population heterogeneity with the experimental results of stress induction experiments and provide predictions about the expected mortality patterns. As biology is moving towards the study of the distribution of individual-based measures, the model and framework we propose here paves the way for evolutionary interpretations of empirical and experimental data linking the individual level to the population level.

## Author Summary

Aging is a widespread phenomenon across the tree of life. From yeast to humans, mortality changes over age have been widely documented. Interestingly, all individuals are not equal with respect to the aging process: large variability in individual life span has been reported, even in clonal populations. Understanding the nature of these differences is of great interest for medical research. So far, two hypotheses have been proposed: individuals may differ in their aging rate or timing. Here, we show that these two hypotheses can reproduce experimental and empirical mortality patterns as a result of natural selection. We also predict the corresponding population heterogeneity in aging. Many studies define subpopulations ad hoc, the work we present provides insight into a more accurate description of inter-individual differences in aging. Finally, our analysis also predicts the modifications of these mortality patterns under stressful conditions. This exploration reproduces experimental data obtained with heat shocks and permits to foresee new mortality patterns that could be observed with other perturbations.

## Introduction

Aging can be generally defined as age-related changes in a set of variables, from growth rate to reproductive effort, which influence the fitness of an organism. Aging is a multiscale process which can be measured at almost every level of the individual organism. Individual metrics of aging include a broad range of processes from damage to DNA and proteins [1], [2] to tissue loss of functionality [3]. Complementary to characterization of aging through individual metrics, there is a long history of demographic studies of aging going back to Gompertz's seminal studies [4]–[6] studying age-specific mortality.

Age-specific mortality is arguably one of the most documented measure of aging. Since Gompertz' seminal work on human data [4], such mortality curves have been obtained for a large variety of species [7], in a broad range of environmental conditions (e.g., [8], [9]). These curves play a central role in understanding aging processes and predicting the dynamics of population growth and human life expectancy [10], [11]. Age-specific mortality curves allow the comparison of aging processes between species as the same measure can be applied from unicellular organisms to humans, as long as the death of the individual is clearly defined. All of these contribute to make changes in mortality over age a well-accepted definition of aging from the demographic perspective. This is the definition of aging considered throughout this paper. One of the striking observations resulting from inter-species comparison is that yeast, fly, worm and human mortality patterns share common properties. They all exhibit exponential increase and decrease with age [7], but also differ in the timing and magnitude of these exponential phases and some species even exhibit mid-age or late-age plateaus [7]. In this article, we study the evolution of mortality patterns to investigate the nature of inter-individual differences in aging.

The nature of inter-individual differences in aging is crucial to study and forecast a population's life expectancy, but is as yet still largely unknown. For humans, two main hypotheses have been proposed recently [11]: individuals may differ in aging rates or timing. Namely, from one individual to the other, the aging processes may be slowed or delayed. Addressing this issue is import to understand the potential and limits of individual medical treatments. Fundamental questions about inter-individual differences, i.e. individual heterogeneity, can be tackled studying age-specific mortality patterns.

Because of these exponential patterns, differences in aging rates and timing can be visualized respectively as changes in slope and shift in mortality patterns [11]. Such changes have been documented for *Drosophila melanogaster* populations: when facing a diet restriction, a change in aging timing occurs while the response to fluctuations in temperature is a change in aging rate [12], [13]. Previous work has used age-specific mortality to show that the aging rate seemed to be conserved in different human populations [14] and differed between different strains of *Caenorhabditis elegans* [15]. Both shift in level and differences in rate have been reported between baboon populations [16] or between male and females flies [17].

These mortality curves are population measures: they result from the aggregation of individual's aging. Biodemographic studies of aging have shown that specific population heterogeneity can reproduce the main features of qualitatively different mortality patterns, such as late-age mortality plateaus [18], [19]. To do so, these studies make ad-hoc assumptions about (i) population heterogeneity, e.g., a Gamma distribution, and (ii) the nature of inter-individual differences in aging. In this paper, we address these issues in the light of evolution, we do not assume a specific population heterogeneity and we explore different types of inter-individual differences in aging.

In our model, we include Gompertz aging, that is exponential increase in mortality hazard with age, and implement heterogeneity in aging rates and timing. Population heterogeneity is allowed to evolve over generations under the influence of life-history trade-offs. Following the disposable soma theory [20], [21] and empirical observations made in a broad range of species [22], we incorporate a trade-off between reproduction and aging. In this framework, investing in maintenance mechanisms (rather than reproduction) results either in slowing or delaying aging depending on the nature of inter-individual differences.

In section ‘Transitions in mortality curves’, we discuss predictions concerning population heterogeneity and the corresponding mortality patterns under different assumptions about inter-individual differences. We compare our results to mortality patterns of yeast, flies, worms and humans respectively. In section ‘Influence of mutation rate’, we test the robustness of our results with respect to mutation rates and highlight new features for the distribution of heterogeneity. Finally, in section ‘The notion of subpopulation’, we exploit the predictions of the model to shed light on the notion of subpopulations which is invoked in numerous experimental studies [23], [24].

## Results

### Age-structured populations

The model we propose describes evolving populations in which individuals age, die and compete for reproduction while they are alive. The offspring they produce fill the next non-overlapping generation until the desired population size is reached. Survival and reproductive success of each individual are connected following the disposable soma theory. The more an individual invests in reproduction, the shorter its lifespan. For each individual, a single parameter 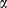 describes its resource allocation strategy between maintenance and reproduction. Different individuals may have different strategies (i.e., different 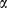) and one of the key features of these models is that this distribution of strategy in the population evolves over generations.

For each generation, all the individuals are synchronized, starting with age zero. While an individual 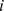 is alive, at each time step it has a probability 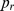 to be chosen for reproduction, proportional to its 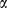 parameter, normalized by the sum of the 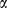 of alive individuals: 
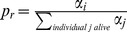
. The normalization factor implements a competition for reproduction because the ability for each individual to reproduce depends on the composition of the whole population. The higher 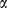 are more likely to reproduce than the lower 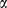, but if the population is reduced to one single individual, the probability it has to reproduce is one, independently of its 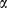 parameter.

Competition for reproduction has been documented in a broad range of species and can take numerous forms, from limited access to habitat [25] to dependence on external resources [26].

Once an individual is chosen for reproduction, it produces one offspring which inherits its 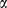 value if no mutation occurs. We use the term mutations here to describe a process introducing variability in the inheritance process. In Text S1, we explore different implementations of such a process and show that the conclusions of this paper do not rely on specific modeling choices. Each reproductive event has a fixed mutation probability 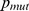: if a mutation occurs, the offspring value of 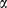 is replaced by a random number drawn from the uniform distribution between zero and one. The result is that 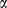 is a heritable trait, subject to mutations and influences the reproductive success of the individual.

The model we propose simulates evolving populations with a fixed size and non-overlapping generations. We start studying individuals at maturity, so that they are able to reproduce since time 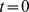. In the initial population each individual is assigned a randomly chosen 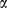 drawn from a uniform distribution between zero and one. We then let population heterogeneity change over generations until it reaches a quasi stationary distribution.

Building the generation 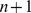 from generation 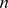 occurs as follows. First, at time 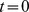, all the individuals are alive. The higher 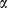 have a higher probability to be chosen for reproduction and therefore the first offspring added to the initially empty generation 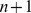 are likely to have high 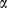. Then, as time goes on, individuals with high 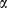 face a faster aging process than their counterparts with low 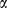. As the high 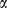 die out, lower 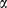 have more and more access to reproduction, thus producing on average low 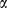 offspring. The ‘low 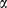
’ phenotype experiences a positive frequency-dependent selection as its fitness increases as their frequency in the population increases. The reproduction process is iterated until the generation 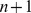 is filled. Some individuals might have inherited a very low 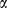 because of the mutation process. They live much longer than the time required to fill a generation and do not reproduce. These individuals we will refer to as the ‘oldest old’ and present the influence they have on mortality patterns in section ‘Transitions in mortality curves’. This reproduction process is asexual, as one individual is either cloned, if no mutations occur, or modified in the other case. In Text S1, we show that introducing sexual reproduction does not alter the conclusions of the paper.

### Choice of the individual mortality function

Following the disposable soma theory, we implement a trade-off at the individual level between survival and reproduction. For each individual, the hazard of death between age 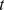 and age 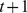 is given by a function which increases with 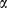 and 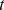. The dependence on 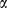 depicts heterogeneity in the population as each individual has its own 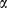. The increase in mortality over age depends on the interactions between (i) the species biological characteristics and (ii) its environment [12], [13]. This interaction is captured by a parameter 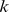 in our model which influences the rate of the individual's exponential aging, differing from one species to the other. This baseline aging is then modulated by the individual strategy of resource allocation between maintenance and reproduction, captured by the parameter 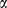. Whether 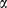 modulates the rate or the timing of the baseline aging is an open question [11]. To address it, we therefore study two distinct individual mortality functions.

First, we implement 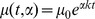 which leads to heterogeneity in aging rates, described afterwards as the Heterogeneity in aging Rate Model (HRM). The parameter 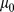 reflects the initial mortality and the parameter 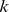 the interactions between the biology of the species and its environment. These two parameters are constant over generations and identical for all the individuals, thus defining a baseline aging.

Second, to derive a mortality function for the Heterogeneity in aging Timing Model (HTM), let us now consider linear dependence on 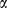 for the individual mortality function and two different individuals, respectively defined by 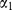 and 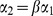. Their respective mortality function 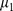 and 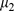 are: 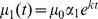 and 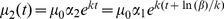. Therefore, a change in initial mortality corresponds to a delay in aging, i.e., a shift of the mortality curve along the time axis, while both individuals still share the same aging rate. In contrast, in the case of the mortality function 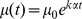 presented in the previous paragraph, two different individuals would start from the same initial mortality but differ in their aging rates.

The two hypotheses concerning heterogeneity in aging postulate different underlying trade-off mechanisms. In the case of heterogeneity in aging rates, investing in maintenance mechanisms slows down the aging process whereas in the case of heterogeneity in aging timing, the same investment delays the aging process. In Text S1, we also present and discuss the influence of age-independent factors, usually referred to as extrinsic mortality, on the evolution of mortality patterns.

### Quasi-stationarity of the evolving populations

Over generations, the evolutionary process reshapes the distribution of 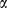-strategies according to the environmental parameter 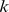 and the choice of the individual mortality function. For instance if 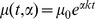, in the (extreme) case of 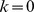, the optimal strategy is 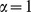 because the trade-off has no effect. On the other hand, high values of 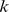 are likely to draw the distribution of 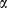 towards lower values so that individuals have to survive to get the opportunity to reproduce. We present the evolution of these distributions in section ‘Evolution of heterogeneity’.

After several hundreds of generations (400 in the simulations below), the distribution stabilizes and mortality curves can be estimated. The probability for a whole population to disappear in the course of evolution is strictly positive. Indeed, each individual has a non-zero probability to die at each time step and this could happen before it reproduces at all. Population size can theoretically decrease over generations until extinction. Yet, for a broad range of parameters, this does not occur and a quasi-stationary distribution of strategies is reached. In Text S1, we provide a mathematical formulation of the model which would allow one to study the existence and uniqueness of these quasi-stationary distributions from a formal standpoint. Here, in the following sections, we focus on the properties of these post-evolution quasi-stationary distributions.

In this paper, we focus on adult mortality patterns and, as such, we do not explicitly address the heterogeneity arising from developmental processes. We assume that development leads to the expression of the inherited phenotype 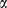. In Text S1, we explore the effects of a maturation period before the onset of reproduction and provide mathematical tools to address the issue of additional development-induced heterogeneity in more depth. This additional information could be used to study the known effects of the environment during development on population heterogeneity [27].

### Transitions in mortality curves

**Figure 1:**
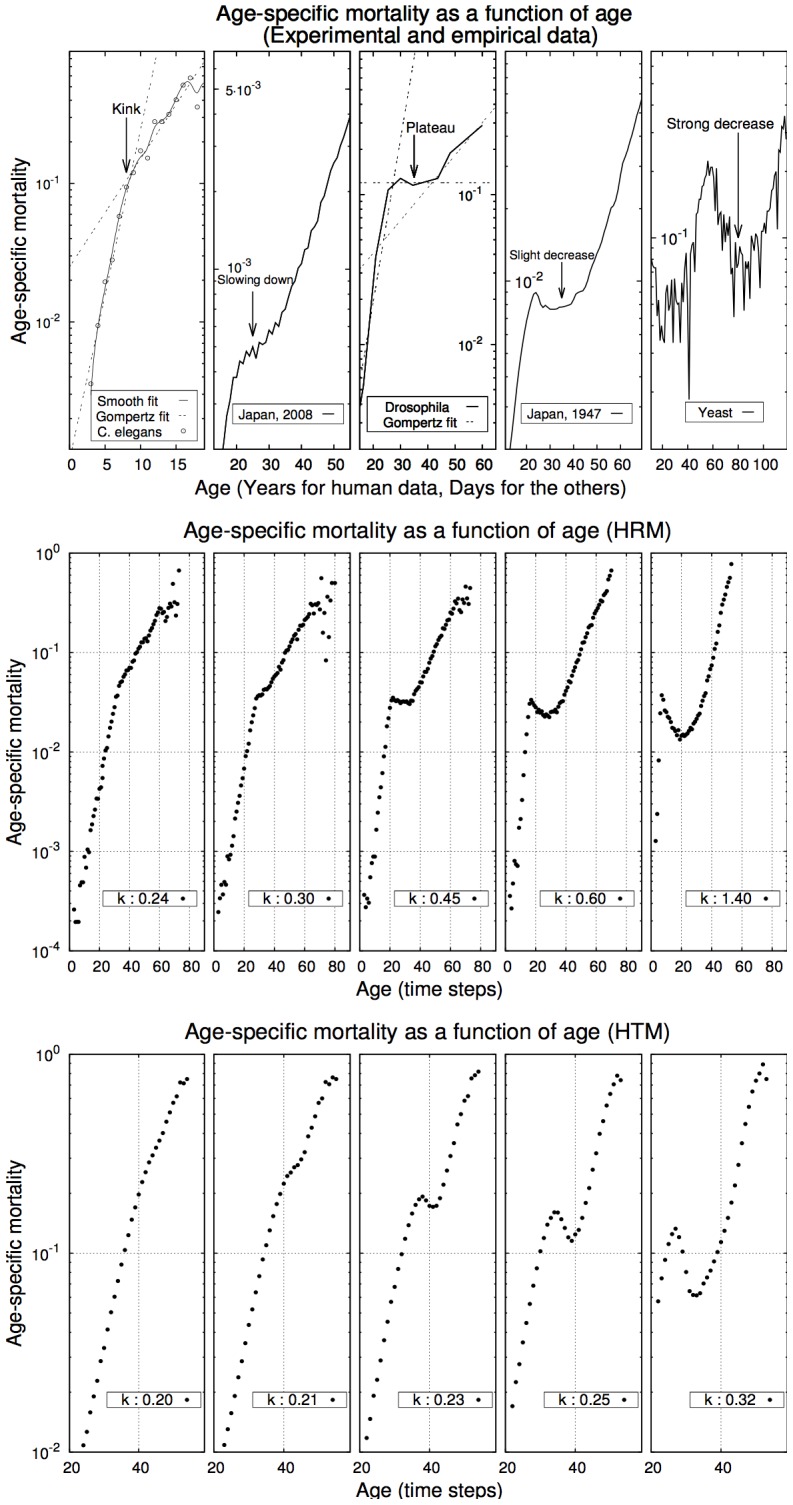
First row, age-specific mortality curves for C. elegans, humans, *D. melanogaster* and yeasts. The qualitative pattern goes from an exponential-exponential pattern to an exponential-U-shape-exponential pattern. Experimental data adapted from Vaupel et al. [7] and [6] for humans.; Second row: Mortality patterns for an individual mortality function 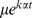 (HRM) in different environments (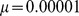, 500 individuals per simulation, 400 generations and 300 simulations). The same transitions as the one depicted in the first row; Third row: Mortality patterns for a mortality function 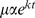 (HTM) resulting from the evolution under different values for 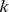 (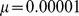, 500 individuals per simulation, 400 generations and 300 simulations per figure). The same transitions as in the first two rows are observed, from an exponential pattern to an exponential-U-shape-exponential pattern.

In this section, we present the results of evolution in different environments under two distinct assumptions, heterogeneity in aging rates (HRM) and heterogeneity in aging timing (HTM), and compare the outcome with empirical and experimental data. In figure 1, first row, we present mortality patterns of C. elegans, D. melanogaster, humans and yeasts. From left to right, the C. elegans mortality curve presents a two-stage exponential increase, usually referred to as a ‘kink.’ Human mortality data also exhibits a two-stage exponential, but is separated by a slowing down or even a slight decrease. The D. melanogaster mortality curve has an exponential-plateau-exponential pattern; in contrast, the one for yeasts display a clear U-shape.

Simulation results with both the HRM and HTM, presented in figure 1, second and third row respectively, exhibit the same set of mortality curves. Both models lead to a transition from an exponential-exponential (‘kink’) pattern under small values for 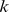 (on the left), to an exponential-slowing down-exponential pattern when evolution occurs under higher values for 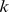. Increasing 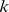 further leads to an exponential-plateau-exponential pattern, then to an exponential-decrease-exponential pattern. Finally, the highest value for 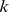 presented here corresponds to an exponential-U-shape-exponential pattern. These transitions in shape reproduce the transitions observed in experimental and empirical data. Obtaining all these experimental patterns only requires the modification of the single parameter 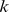 and letting population heterogeneity evolve.

In Text S1, we show that these mortality patterns are also robust to an extensive exploration of parameter space.

**Figure 2:**
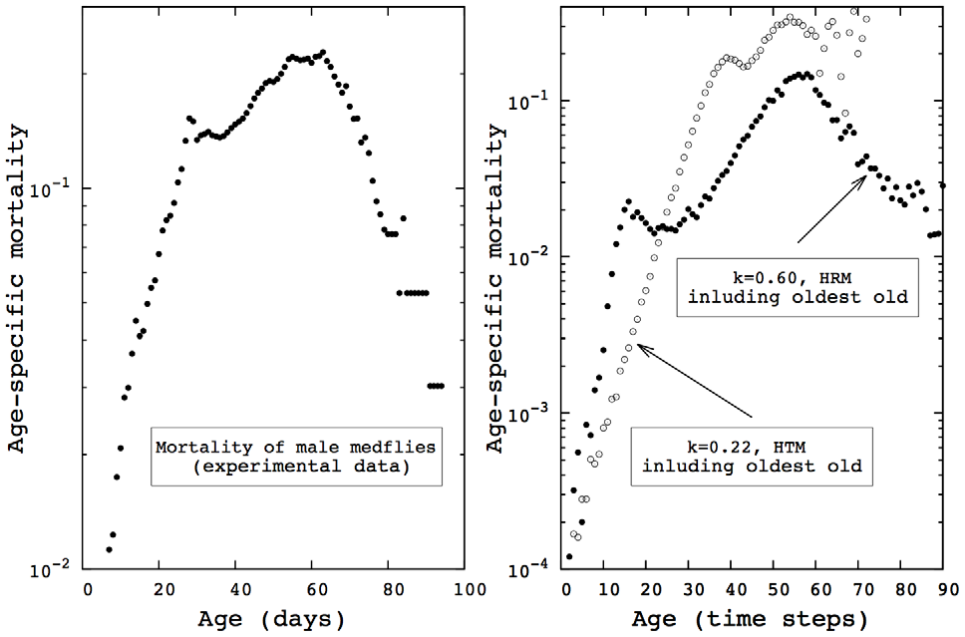
Both the HRM and the HTM reproduce the key features of medfly mortality pattern: (i) increase, (ii) local maximum, (iii) U-shape, (iv) second local maximum, higher than the first one, (v) decrease. The scales of the y-axis differ in both graphs in order to zoom around the region of interest. (Experimental data adapted from Vaupel et al. [7], 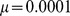, 500 individuals per simulation, 400 generations and 300 simulations per curve).

The transitions in mortality patterns presented in figure 1 are calculated without accounting for the oldest-old individuals, i.e., those living longer than the time it takes to fill a generation. In figure 2, we present the influence of these individuals on mortality patterns. Including these individuals does not modify the qualitative shapes presented in figure 1 but leads to a decrease in mortality at late ages. Simulated mortality patterns of both the HRM and the HTM resemble medfly mortality which presents two peaks and a late-age decrease.

### Evolution of heterogeneity

**Figure 3:**
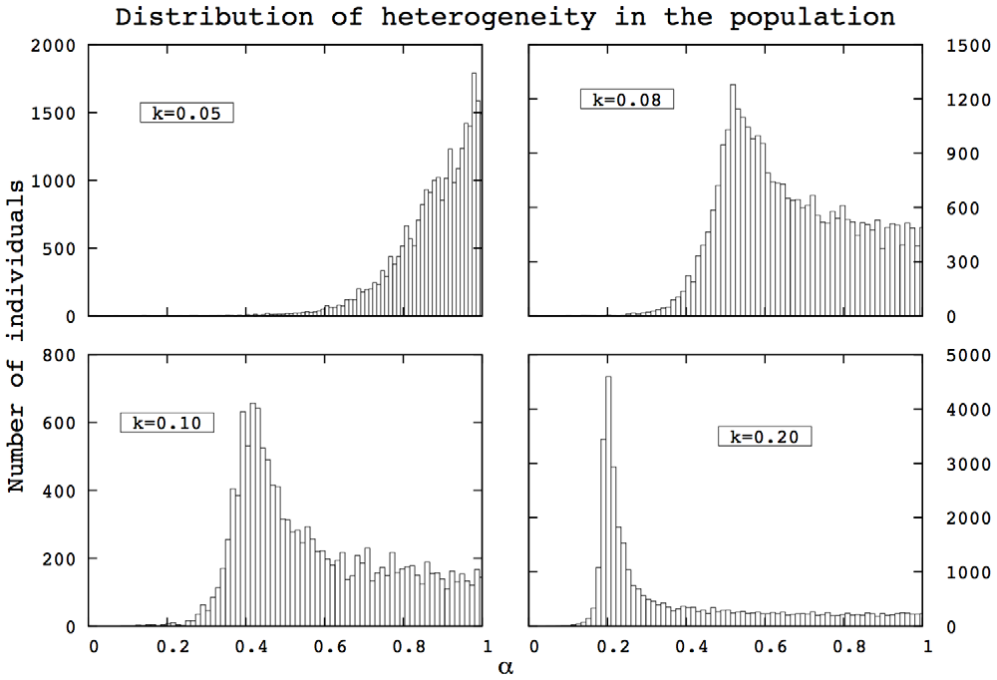
Population heterogeneity depends on the interactions between the species biology and its environment. These interactions are captured by the parameter 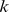. The resulting distributions of heterogeneity for an exponential frailty model are presented above, corresponding to the mortality curves presented in figure 1, second row. 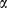 represents the investment in reproduction made by the individual. Higher basal damage accumulation (high 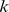) leads to more individuals investing in maintenance (i.e., low 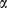 due to life-history trade-off). In both this figure and figure 4, the scale of the y-axis is set to match the highest frequency.

In both models, population heterogeneity is shaped by natural selection, via a life-history trade-off between survival and reproduction. The transitions observed in the two versions of the model rely on a change of the underlying heterogeneity in 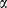 in response to the parameter 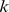. Figure 3 shows the quasi-stationary distribution of 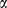 as a function of 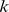 in the case of heterogeneity in aging rates. Under low basal damage accumulation (low 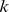), the most prevalent strategy consists of investing in reproduction (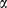 close to one). As 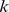 increases, being able to survive in order to reproduce leads the distribution of strategy to move towards more investment in maintenance mechanisms (low values of 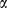). Mortality patterns presented in figure 1, second row, directly derive from these evolved distributions of heterogeneity.

**Figure 4:**
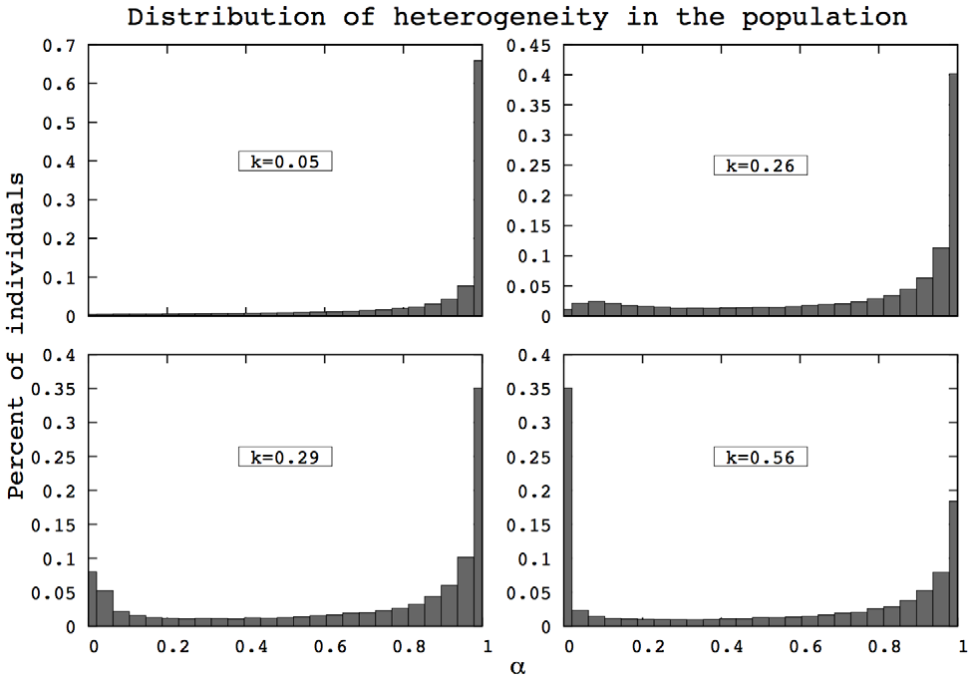
Distributions of heterogeneity, for a mortality function 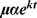, underlying the mortality curves presented in **figure 1**, third row. With heterogeneity in aging timing, bimodal distributions emerge from the evolutionary algorithm. Only the ratio between the two peaks changes with the environment.

In the case of heterogeneity in aging timing, population heterogeneity evolves with the parameter 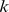 as depicted in figure 4. The same qualitative understanding holds: under low damage accumulation, investment in reproduction is favored while maintenance becomes prevalent if damage accumulates faster. Here, the distributions are bimodal and increasing 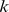 mainly changes the ratio between the two peaks, while also slightly shifting the left peak towards more maintenance. In the previous case (heterogeneity in aging rates), the distributions are unimodal and the main effect of 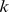 is the shift.

In our models, individuals are competing with each other to access reproduction. Different 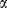, that is different resource allocation strategies, have different prevalence, as shown in figures 3 and 4. As the parameters 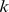 and 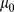 are fixed initially and kept constant over generations, the fact that a whole distribution of strategies is maintained in the population, without one 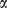 taking over, could be surprising. Previous models of competition have shown that in many set-ups, one strategy invades the population and the distribution of heterogeneity after several generations is a single peak corresponding to the optimal strategy or species [28]. Polymorphism can be maintained in density-dependent selection models, but it usually requires negative density-dependence [29] (that is the fitness of a trait increases as the frequency of the trait in the population decreases). Interestingly, in this model, heterogeneity is maintained over generations even though all the 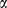 experience a positive density-dependent selection. In Text S1, we account for this sustained heterogeneity in both the HTM and the HRM.

### Influence of mutation rate

Mutations occurring on 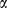 influence the heterogeneity distributions in evolved populations. A high mutation rate tends to make the distribution of 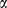 closer to a uniform distribution. In this section, we first present the effects of high mutation rates on populations heterogeneity as well as the corresponding effects on the mortality patterns.

**Figure 5:**
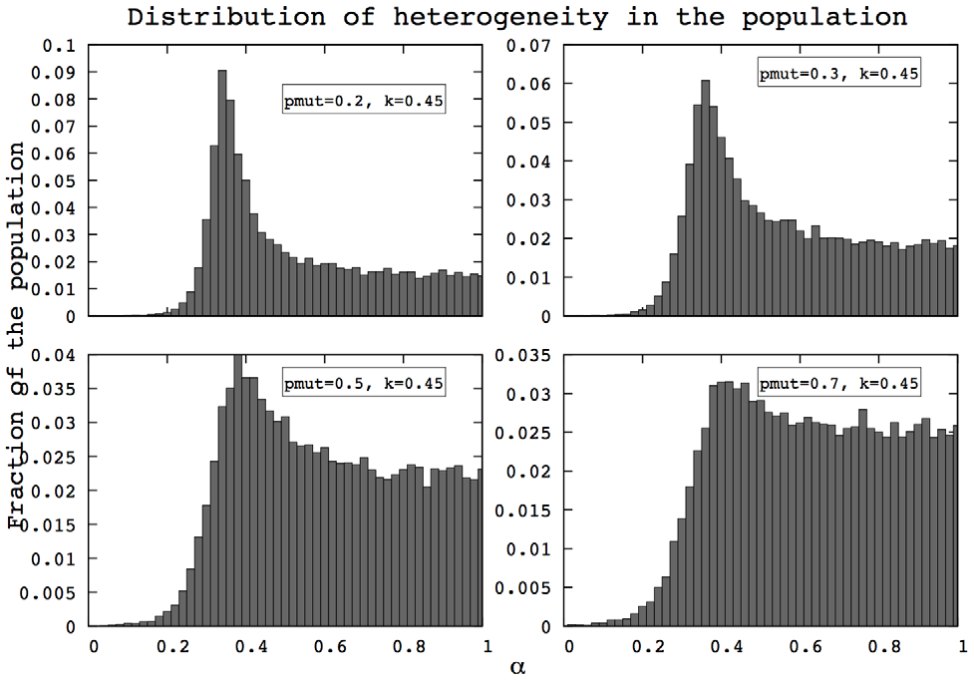
Distributions of 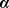 after 400 generations in the HRM for different mutation rates. The left part of the distribution remains unchanged, while the right tail gets closer to a uniform distribution as the mutation rate (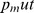) increases. (HRM, 400 generations, 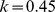, 500 simulations).

**Figure 6:**
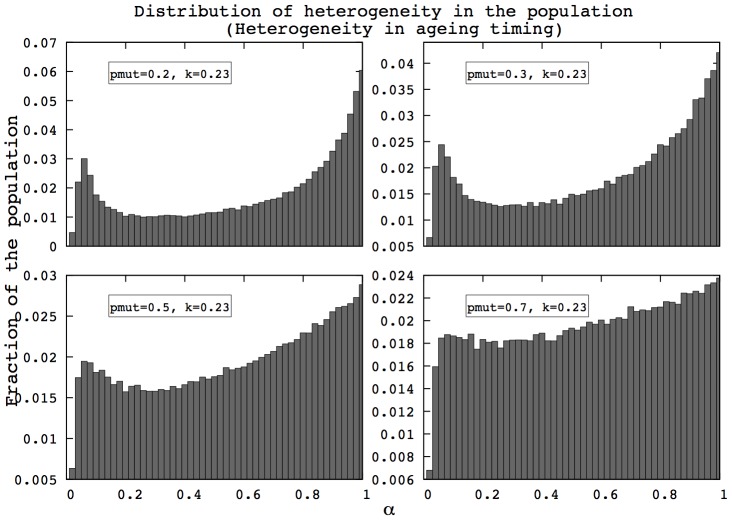
Distributions of 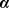 after 400 generations in the HTM for different mutation rates. The left part of the distribution remains unchanged, while the right tail gets closer to a uniform distribution as the mutation rate (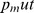) increases, which is similar to the observation in figure 5. (HRM, 400 generations, 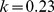, 500 simulations).

First, we find that the properties of asymmetry in heterogeneity described in figure 3 and 4 are maintained despite high mutation rates, as shown in figures 5 and 6. The left side of the distribution is not altered by the mutation process: the shape of the distribution close to zero remains unchanged even under high mutation rates. This derives from the fact that individuals below a certain threshold have negligible opportunities to reproduce and are therefore absent from the population. More details about the asymmetry between low 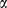 and high 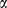 are provided in Text S1.

**Figure 7:**
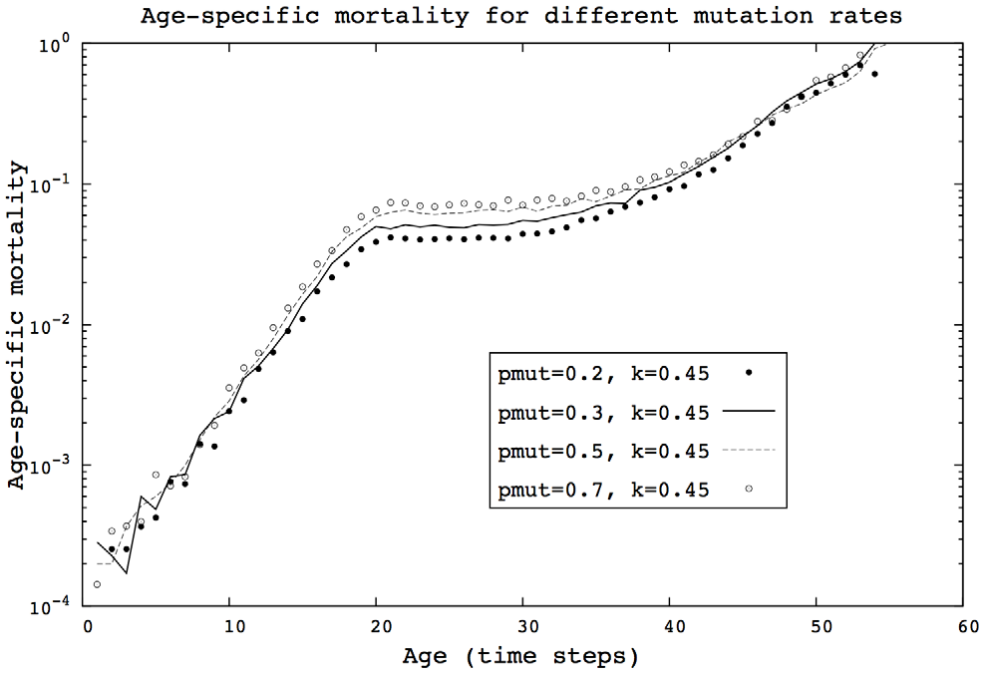
Even with high mutation rates, the mortality patterns are not significantly altered in the HRM model. (HRM, 400 generations, 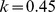, 500 simulations).

**Figure 8:**
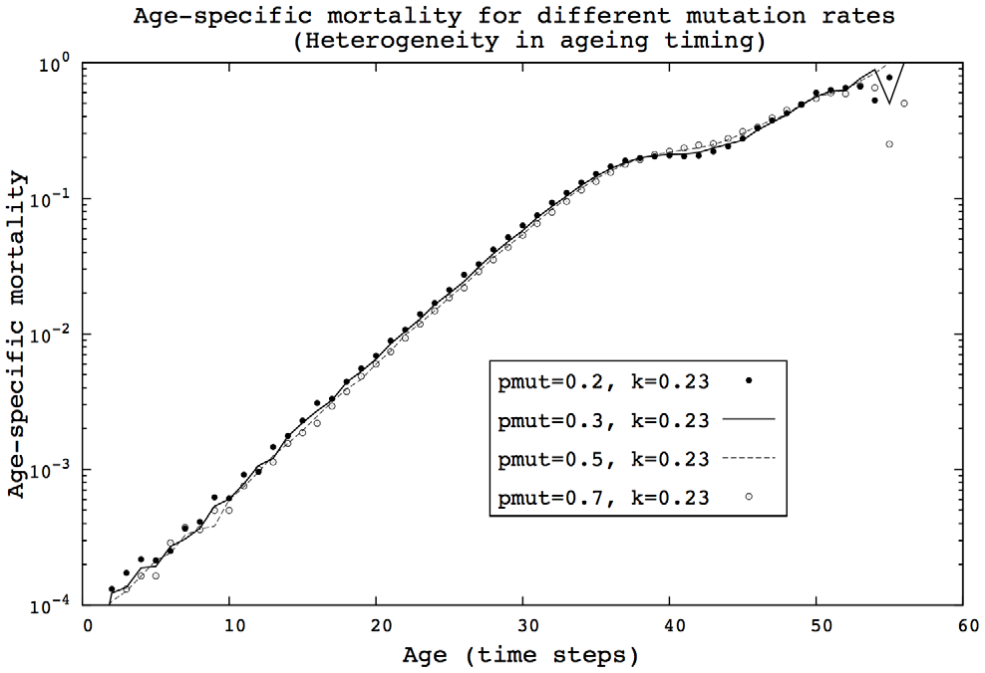
Even with high mutation rates, the mortality patterns are not significantly altered in the HTM model. (HTM, 400 generations, 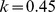, 500 simulations).

Results in sections ‘Transitions in mortality curves’ and ‘Evolution of heterogeneity’ suggest that changes in population heterogeneity have a considerable influence on mortality patterns, providing the transitions in shape previously described. Yet, we find here that if the left-side of the 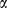 distribution is preserved, then mortality patterns are not qualitatively changed, as shown in figure 7 and 8. In this case, the exponential-plateau-exponential pattern remains unchanged. These findings imply that the presence of the strongest individuals dramatically influences the shape of mortality patterns.

### The notion of subpopulation

#### Defining subpopulations

**Figure 9:**
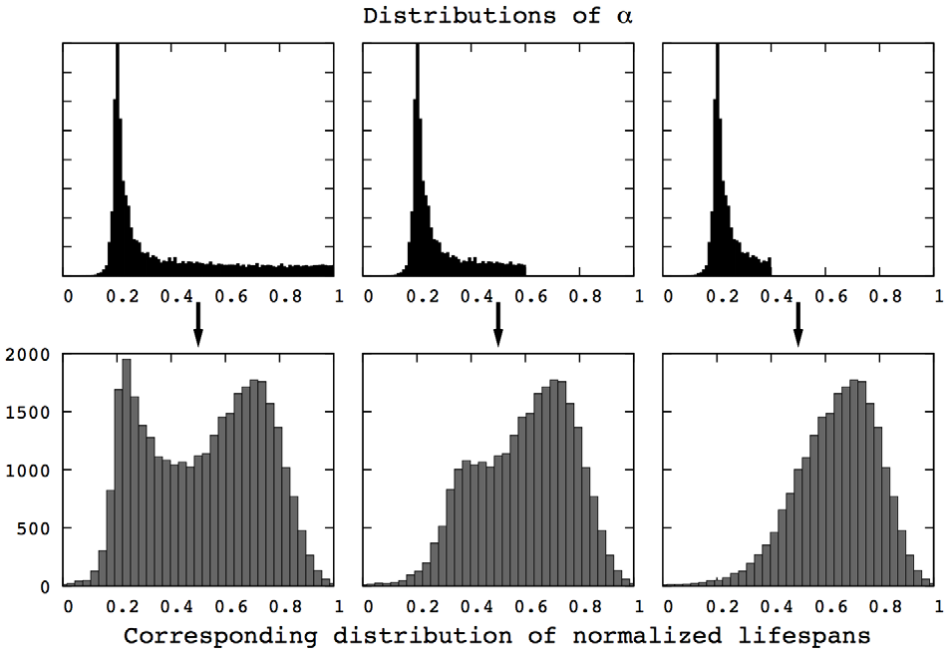
A bimodal distribution of lifespan does not necessarily imply the existence of two subpopulations with respect to an aging marker. If the link between this aging marker and mortality is non-linear, then a unimodal and long-tailed distribution can lead to a bimodal distribution of individual lifespans. In the HRM, all the individuals in the right tail of the distribution contribute to the same peak in the distribution of lifespan (HRM, 400 generations, 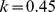, 500 simulations to obtain the uncut distribution of 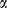).

Because it explicitly links the individual level and population measures of aging, the model we propose also allows for study of what is called ‘subpopulations’ in several experimental studies. In the case of heterogeneity in aging rates, the distribution of individual lifespan in evolved populations is bimodal for 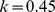 as shown in figure 9, first column. Yet, the corresponding distribution of 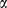 is unimodal. Therefore, the very notion of subpopulation seems to be challenged here. Indeed, observing the lifespans leads to the distinction of two subpopulations which cannot be easily determined when analyzing the distribution of 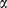.

When the right tail of the 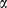 distribution in the HRM is cut-off, the bimodality in the lifespan distribution progressively disappears (figure 9, all columns). All the individuals in the tail contribute to the same peak of the lifespan distribution. This phenomenon is due to the non-linear relationship between 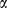 and lifespan which allows the compression of a broad range of 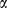 values onto a single lifespan peak. In terms of experimental study, if 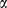 is the chosen marker to assess investment in maintenance, the findings we present here show that the knowledge of the relationship between this marker and the related aging feature is key. If it is strongly non-linear, defining subpopulations is difficult.

#### Subpopulations in stress induction experiments

A subpopulation also arises in studies focused on stress induction experiments [24]. The usual set-up consists of measuring a marker linked to survival, such as concentration of chaperone proteins, and splitting the population into several subgroups, very often two: high expression and low expression profiles. As mentioned above, the existence of subpopulations may depend on the marker which has been chosen, the key being the nature of the relationship between this marker and the aging criteria observed (such as lifespan).

**Figure 10:**
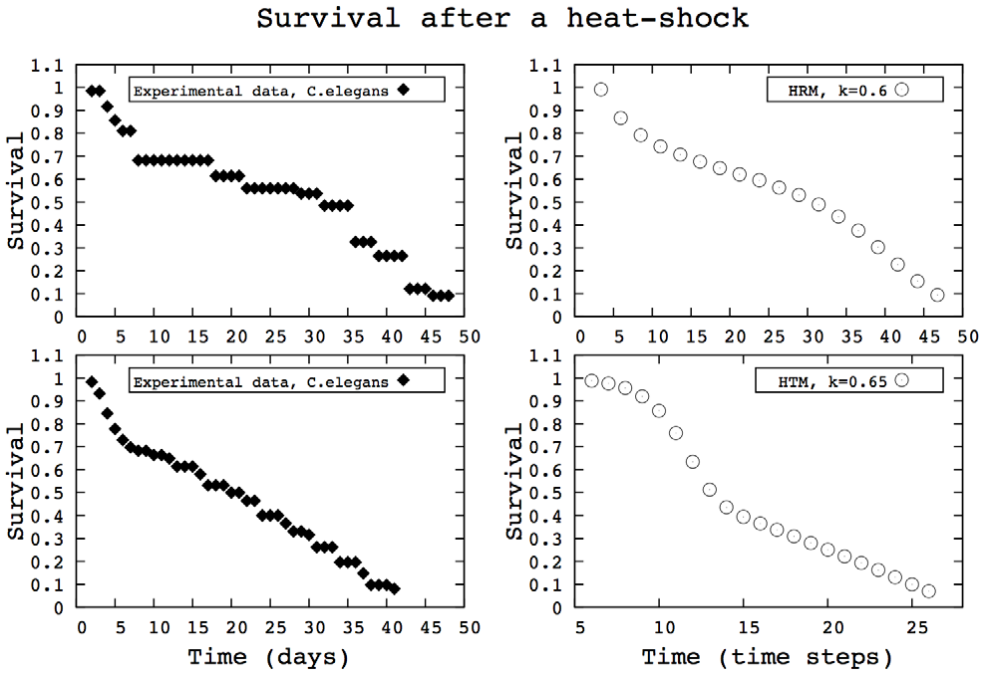
Survival curves after a heat-shock with heterogeneity in aging rates (HRM) and heterogeneity in aging timing (HTM), on the right column. The two curves reproduce the main features of survival curves corresponding to heat-shock experiments in C. elegans (filled diamonds, adapted from [24]). Both the experimental data and the simulated curves exhibit a two stage decrease: a first quick, strong fall followed by a slowing down. (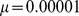, 500 individuals per simulation, 500 simulations per curve).

As a case study, we focus here on stress induction experiments in C. elegans. Figure 10, left column, shows the survival curves for populations of C. elegans after transient heat-shocks. The main features of these survival curves are characteristics of stress induction experiments: the survival curve exhibits a strong decrease, followed by a slowing down. Such patterns can be found in a broad range of species and experimental conditions [30]–[32]. The right column of figure 9 shows two survival curves which correspond to populations evolved with heterogeneity in aging rates and heterogeneity in timing respectively. Both models reproduce the key features of the experimental data of [24]. In this paper, the authors hypothesized the existence of two subpopulations (‘strong’ and ‘weak’ individuals) to account for their experimental results. Here, we show that both unimodal and bimodal distributions of population heterogeneity lead to similar survival pattern, depending on the relationship between 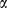 and the mortality function.

### Predicted mortality patterns in stress induction experiments

**Figure 11:**
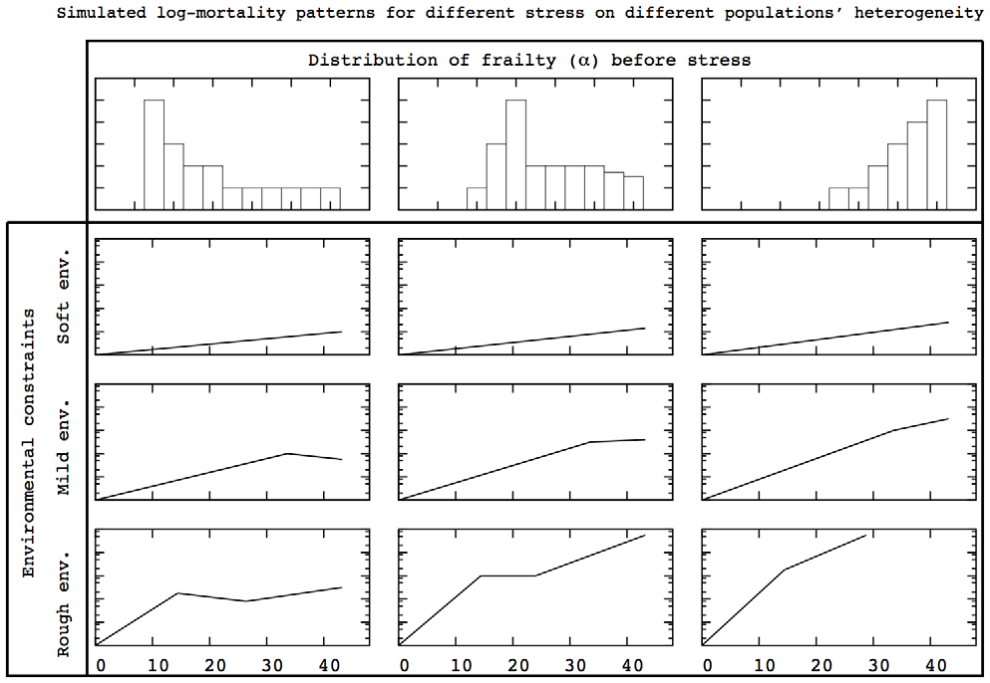
Expected log-mortality patterns in stress induction experiments for the HRM. Modifying 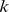 without adaptation in population heterogeneity changes the mortality pattern. The results presented in the right column are in agreement with experimental results concerning diet changes in C. elegans [9].

**Figure 12:**
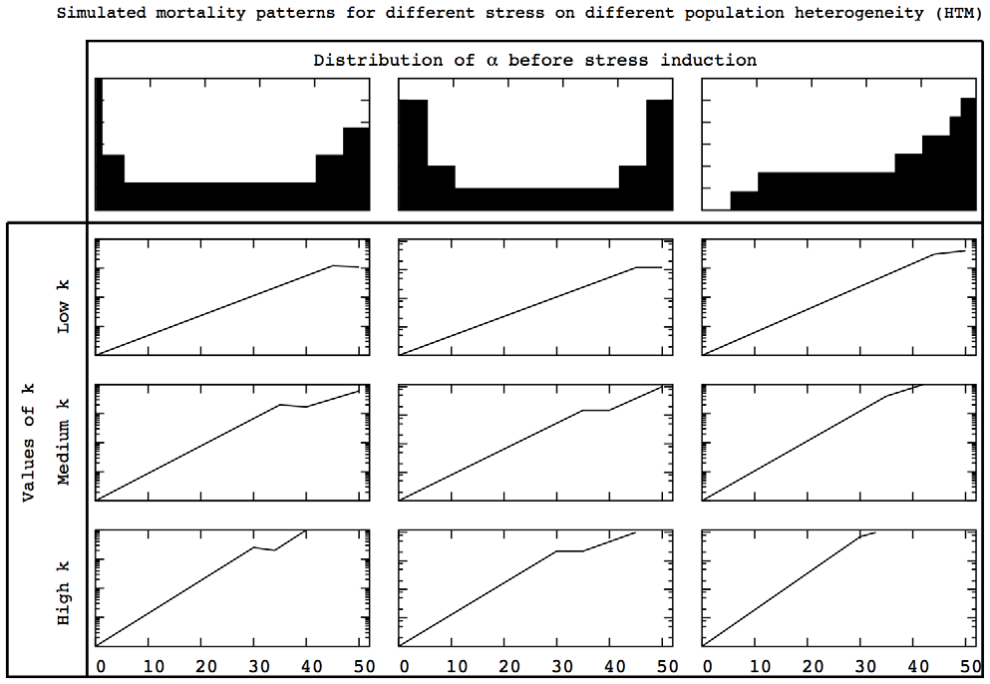
Expected log-mortality patterns in stress induction experiments for the HTM. Modifying 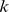 without adaptation in population heterogeneity changes the mortality pattern. The results presented in the right column are in agreement with experimental results concerning diet changes in C. elegans [9].

Whether stress induction experiments induce adaptation which modifies population heterogeneity is a burning issue [27]. If adaptation occurs when 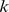 is modified, mortality patterns presented in figure 1 emerge. In this section, we complement the previous results with predictions concerning the mortality patterns expected when there is no adaptation of population heterogeneity. In figures 11 and 12, we show the qualitative changes in mortality patterns when 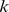 is modified but not the distribution of 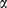 in the HRM and the HTM respectively.

In both figures, the distribution of 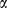 presented in the right column almost always led to exponential-exponential (kink) mortality patterns. The only exception occurs for the HRM and high 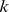 combination because individuals die too quickly to exhibit the two-stage exponential increase. In all the other cases, the kink shape is preserved but the time at which the kink occurs as well as the corresponding mortality levels are modified by 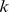. These findings are in agreement with experimental results for C. elegans observed under different diets [9].

The results presented in the other columns provide predictions concerning the expected mortality patterns for other population heterogeneity. The interests of this exploration are twofold. First, it allows one to determine whether a given stress induction experiment will modify population heterogeneity. If the same distribution of 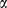 cannot produce the mortality pattern with and without stress by only modifying 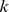, our model predicts that adaptation as occurred. Second, if adaptation does not occur, the mortality patterns under perturbation present significant differences between the HRM and the HTM. One of the main observation which stands out from the comparison between figure 11 and 12 is that the mortality rates are more modified in the HRM than in the HTM in response to changes in 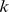. Therefore, this prediction can provide a way to decide whether inter-individual differences in aging rely on rate of timing.

## Discussion

Studying the evolution of heterogeneity in resource allocation strategies is at the heart of a broad range of experimental studies and today's aging research [11]. The nature of individual mortality functions are still to be discovered: whether populations of the same species living in different environments differ in aging rates or aging timing is still an open question. Drosophila melanogaster submitted to diet changes exhibit a shift in their mortality curves (changes in the timing) while the same populations exhibit a rescaling of their mortality curves in response to temperature changes (changes in aging rates) [13]. The same question holds for human populations and individuals [11]. Understanding the nature of heterogeneity in aging sheds light on the biological constraints which can be circumvented. If it turns out that aging rate is the same for every human being, it might be strongly controlled at the lower levels of the organism and therefore difficult to modify.

Moreover, in terms of medical treatments, focusing on aging rate or timing has distinct implications. On the one hand, modifying the rate of aging results in a high potential increase in life expectancy as aging is slowed during the whole life time. On the other hand, such changes in rate would not only extend the period of ‘healthy aging’ but also morbidity. These pros and cons have arguably significant implications in terms of public policy and need to be considered with regards to population heterogeneity. If the rate of aging is a fundamental constant in humans while timing is highly variable, being able to modify aging rate via medical progress will require substantial investment while timing would be presumably more flexible and thus cheaper to modify.

Numerous studies in current aging research have found mutations or treatments which dramatically influence aging dynamics [12], [13]. Yet, very often the issue of timing and rate of aging is not directly addressed, for instance because survival curves are provided and not mortality curves. The models we present in this paper allow one (i) to compare their data to the transitions with the parameter 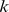 observed in our model and (ii) to reinterpret previously published data to address the crucial issue of rate and timing of aging.

In this model, we analyze different possible heterogeneity in aging, only assuming Gompertz dependence on time. Our results show that assumptions about internal life-history trade-offs modulate the shape of population heterogeneity. Yet, in a wide array of scenarios, the same set of mortality and survival curves emerge. Population heterogeneity adapts to the environment according to the individual mortality functions but, at the population level, no qualitative changes can be observed. The transitions observed always go from a ‘kink’ shaped mortality curve to an exponential-U shape-exponential pattern. This robustness of the mortality patterns also echoes experimental observations of mortality and survival in different environments. In many organisms, the modifications observed in mortality patterns as a result of genetic manipulations [33] or environmental changes [9] does not provide strikingly different curves. These curves can be stretched or shifted by the treatment, but generally they do not exhibit shapes radically different than those described in this paper. For instance, the survival curve shown in figure 10, which shows a quick decrease followed by a slowing down, is a standard pattern which can be observed across species under stressful conditions [30]–[32]. This robustness of survival and mortality curves will be easier to assess thanks to increasingly available data for species other than model organisms. Such robustness may even be a key property which sheds light on the underlying mechanisms of aging across species, their effects, their limits and their evolution.

The systematic exploration of different forms of inter-individual differences in aging also provides predictions about the expected distributions of heterogeneity. Even though the mortality patterns look alike in the different versions of the model, the evolved heterogeneity distributions do not. Current technical advances, such as microfluidics set-ups [34], individual genomics [35] or proteomics [36], allow more and more observations at the individual level, which in turn lead to population level influences. As such, we expect that the analysis of distributions and heterogeneity will replace the analysis of means over populations in the coming decade. The framework we present here, in which the distribution of heterogeneity conveys information about evolution and the aging process, anticipates this trend and paves the way for formal modeling and understanding of individual-based data. In aging research, these predictions would allow researchers to decide whether individual differences in aging rely on aging rate or timing. In other fields, it paves the way for a renewed vision of inter-individual differences evolution.

Previous works have used the Price equation to link population heterogeneity and natural selection [37], [38]. The framework we propose here, with age-dependent competition and evolution of distribution provides an interesting ground for further development of these works. In this respect, the analytical framework presented in the Methods section is of particular interest as it could in principle give explicit expressions concerning the changes in the phenotypic variance from one generation to the other.

Moreover, the shape of these distributions also provides information about the link between the marker observed at the individual level and the measure of interest, such as individual lifespan. Comparing the distributions emerging from our evolutionary algorithm shows that to obtain the exponential-plateau-exponential pattern (which corresponds to a bimodal distribution of lifespans) with a unimodal distribution of the aging marker, the link between the marker and individual lifespan needs to be strongly non-linear. The exploration we propose here also provides milestones to interpret the individual-based data and their corresponding distributions.

Finally, we believe that the model is also flexible enough to allow the exploration of other types of life-history trade-offs, based on the key idea of evolving heterogeneity. As such, it paves the way for future interpretations of coming individual-based observations in evolving biological systems.

### Conclusions

The simplicity of the framework we propose also enables the formalization of intuitive notions, such as a ‘subpopulation’ and the extensive exploration of mortality functions provides predictions of possible curves for organisms yet to be studied, along with expected distributions of heterogeneity. The robustness of mortality patterns observed suggests that aging is itself a robust process, relying on similar processes across species. We hope that this work paves the way for (i) faster understanding and classification of heterogeneity distributions across species which are not model organisms and (ii) opens up new prospects in terms of understanding the evolution of aging and its robustness. The set-up allows easy changes and explorations, as well as creating space to make the interactions between aging and reproduction more complex. In that sense, it complements previous approaches combining evolutionary theories and heterogeneity, as it provides a framework to explore yet to be explained aging dynamics.

## Methods

### Numerical methods

We have simulated our model with both a continuous time and a discrete time framework, in both a stochastic and a deterministic manner, in order to ensure the robustness of our results. All models have been implemented in C and this section provides numerical methods for the algorithm used in our simulations.

#### Stochastic models

In the stochastic models, the population has a finite size which is usually 500 individuals, unless otherwise mentioned. Modifying this parameter does not alter the conclusion of the models and the same results can be obtained for different population sizes. The mortality curve we observe as a result of the evolutionary algorithm depends on the parameter 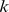. The transitions in the mortality patterns following 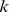 are the same for different population size, these transitions simply occur for different values of 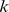.

In the discrete time version of the stochastic model, the age of each individual is incremented by one at each time step and followed by a survival test. This test consists of drawing a random number 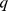 between zero and one, and comparing this number with the age-specific mortality hazard: if 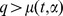, the individual survives until the next time step and dies otherwise. As we consider populations with a fixed size of 500, no individuals were experiencing a mortality hazard higher than one. As a first approximation, we have considered the age-specific probability of death to be close to the mortality hazard at the same age. This approximation provides the same results as the continuous-time version of the model (see below) which directly computes the individual lifespan according to mortality hazards. All alive individuals compete then for reproduction. Multiple reproductive events may occur in the same time step, and the offspring produced is added to the next generation if it is not full. This process of death-reproduction is then iterated until the next generation is filled.

The continuous time version of the stochastic model follows the same principles. The time of death and reproduction events are drawn following continuous distributions. First, the time of death for each individual is randomly drawn following an exponential distribution which corresponds to its 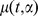.More precisely, the procedure to draw the time of death 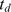 for a given individual, with a parameter 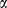 and a baseline mortality described by 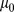 and 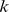, is the following. First, we draw randomly a number 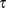 between 0 and 1. Then, we compute 
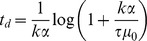
, following the standard procedure to draw random numbers from exponential distributions. The time of reproductive events derive from a standard Gillespie algorithm accounting for the fact that only alive individuals compete for reproduction. More precisely, the time interval between two reproduction events is drawn from an exponential distribution of parameter 1, as these are the only events considered here (the time of death being already calculated beforehand). The stochastic model with continuous time was used for all the figures presented in the main text.

### Deterministic model

We have also implemented a deterministic model which corresponds to the stochastic models presented in the main text in the case of infinite populations. The purpose is twofold: (i) it ensures the robustness of our results and (ii) it paves the way for an analytical analysis of the evolutionary algorithm we present in this paper. For instance, the formalisation presented in this section allows one to study from a formal standpoint the convergence to a stationary distribution with infinite populations. The deterministic model is defined at the population level and describes the changes in 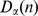, the distribution of 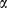 in the population at generation 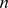. This distribution changes over generations through the same principle: individuals reproduce based on their 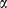 and die according to the individual mortality function chosen. The distribution of 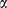 changes over time in one generation, as individuals die, following: 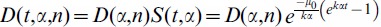. 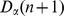 is therefore defined as
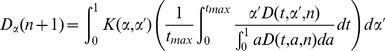
where 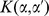 accounts for the probability that an offspring inherits a parameter  given that its parent had a parameter 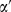 and 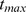 for the finite size of the population. Indeed, as the rate of reproduction is constant over time, limiting the population size is equivalent to limiting the overall time allowed for reproduction. In sum, 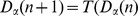 where 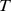 is the operator on 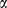 distributions described in the equation above. The evolutionary process described in the main text converges towards a stationary distribution of 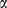 after several hundreds of generations when a fixed point for the operator 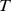 is reached. In Text S1, we show that the deterministic model and the stochastic models produce the same results.

#### Deterministic model

For computational reasons, the possible values of 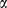 are discrete. In the formula concerning the deterministic model presented above, the integrals with respect to 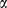 are discrete. The stochastic model with discrete time is equivalent to a deterministic model with discrete time. In this case, each time step follows the same pattern as the stochastic model: survival test ends reproduction. These two steps occur at the population level for the deterministic model with discrete time. A fraction of each class of 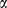 survives and reproduction depends on the number of individuals in each class of 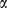.

In supplementary material, we also describe the explorations mentioned in the main article (Text S1) and provide the code to reproduce the simulation results (Program S1).

## Supporting Information

Figure S1Exploration of the parameter space A - Mortality pattern for a mortality function 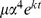 for different values of 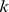 (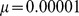, 500 individuals per simulation, 400 generations and 300 simulations). Similar patterns are observed for other powers of 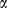. B - Mortality pattern for a mortality function 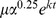 for different values of 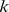 (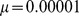, 500 individuals per simulation, 400 generations and 300 simulations). Similar patterns are observed for other powers of 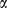. These two new mortality functions provide the same set of mortality curves as those presented in figure 1, main text. C - Mortality pattern for a mortality function 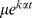 for different initial mortality (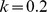). D - Mortality pattern for a mortality function 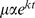 for different initial mortality (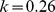). Modifying the initial mortality does not provide qualitatively new mortality curves: the same transitions when changing 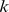 are still observed.(EPS)

Figure S2HRM where mutations consist in perturbing the previous value of 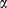. The new value is drawn following a Gaussian distribution, centered on the previous value and with standard deviation 0.3. The same transitions as the model presented in the main text are observed.(EPS)

Figure S3In the HRM, adding mild extrinsic mortality (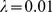) does not alter the conclusions of the paper: the same transitions occur. The main difference lies in a plateau at early ages, resulting from extrinsic mortality driving population death. At mid and late ages, intrinsic mortality dominates and the usual patterns are obtained.(EPS)

Figure S4Transitions with the 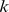 parameter for the deterministic model: the same set of transitions as those presented in figure 1 (main text) emerge from the evolutionary algorithm.(EPS)

Figure S5Mortality pattern and stationary distribution obtained with sexual reproduction. The horizontal line shows 0.01 to highlight the decrease of the tail close to 1. (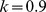, 500 simulations, 500 individuals in each, 400 generations with the HRM).(EPS)

Figure S6Mortality pattern and stationary distribution obtained with a maturation time of 10. The horizontal line shows 0.01 to highlight the decrease of the tail close to 1. (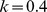, 500 simulations, 500 individuals in each, 400 generations with the HRM).(EPS)

Figure S7In the absence of mutations, the distribution of 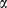 quickly converges towards a peak around one specific value (Heterogeneity in aging rates, 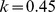, deterministic model). The depletion of high 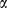 over generations occurs more slowly than the depletion close to zero.(EPS)

Figure S8For small mutation rates, population heterogeneity remains bimodal in the case of heterogeneity in timing (HTM). Heterogeneity is maintained because of the time-dependent competition, as described in the mathematical model in Text S1, section “Maintaining heterogeneity” (HTM, 400 generations, 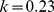, , 500 simulations).(EPS)

Figure S9The grey area represents the set of valid couple 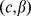 to allow the coexistence of three competing traits in the simple model we present here.(EPS)

Program S1 Simulation Code. Program S1 contains a C code allowing to reproduce the mortality curves of the paper.
(ZIP)

Text S1 Exploration of the parameter space and mathematical models. Text S1 describes supplementary information mentioned in the main text, such as exploration of parameters to ensure the robustness and mathematical models to illustrate how heterogeneity can be maintained over generations.
(PDF)
