# Supplementary material for: How Evolving Heterogeneity Distributions of Resource Allocation Strategies Shape Mortality Patterns
Source: PLoS Comput Biol. 2013 Jan 17;9(1):e1002825. doi: 10.1371/journal.pcbi.1002825 (PMC3547821; doi:10.1371/journal.pcbi.1002825)
Supplement: Text S1 — Exploration of the parameter space and mathematical models. Text S1 describes supplementary information mentioned in the main text, such as exploration of parameters to ensure the robustness and mathematical models to illustrate how heterogeneity can be maintained over generations. (PDF) [file pcbi.1002825.s011.pdf]

Exploration of the parameter space

In order to assess the robustness of the results presented in the main text, we have performed an extensive exploration of the parameter space. Modifying further the mortality functions does not provide qualitatively new mortality patterns, as shown in figure S1, A and B. Changing the initial mortality (μ_0) in the HRM and the HTM does not alter either the conclusions of the model: a limited set of mortality curves emerge from the evolutionary algorithm as shown in figure S1, C and D. As detailed below, we have also run simulations (i) modifying the mutation process on α and (ii) including extrinsic mortality. These two other versions of the algorithm also lead to the same conclusions as those presented in section "Transitions in mortality curves" (main text). Finally, the equivalent deterministic model described below also yields the same conclusions that the models presented in the main text.

- Polynomial dependency on α: $\mu(t, \alpha) = \mu_0 \alpha^n e^{kt}$ where n is an integer, bigger than one.
- Concave dependency on α: $\mu(t, \alpha) = \mu_0 \alpha^a e^{kt}$ where a is a real number, strictly between zero and one.

Different mutations for α : In the different versions of the model presented in the main text, the effect of a mutation on α is to assign a new random value, drawn uniformly between zero and one. Here, we present the same model where mutations consist in perturbing the previous value of α. More precisely, if a mutation occurs, the new value for α is drawn following a Gaussian distribution, centered around the previous value. We have implemented different standard deviations, and none of these versions lead to different conclusions than the models presented in the main text (see figure S2 for a standard deviation of 0.3).

Addition of an Extrinsic mortality : Extrinsic mortality is simulated as a source of mortality independent of age and α. In terms of numerical implementation, an expected time of death is drawn according to the method described in the Methods section (main text) for the intrinsic mortality and another for the extrinsic mortality (from an exponential distribution with a constant parameter λ). The smaller of the two times is taken

to be the lifespan of the individual. Figure S3 shows the transitions in mortality curves for $\lambda = 0.01$.Changes in λ over generations have different effects. On the one hand, if λ is varying slowly compared to the convergence of population heterogeneity, say changes every 50 generations, then one would observe that the distribution of α is converging from one stationary distribution to the other. On the other hand, if λ is varying quickly, say between two values at every generation, then the resulting distribution of α is a mixture of the two stationary distributions.

Deterministic model : As stated in the main text, the deterministic model produces the same transitions in mortality curves as the stochastic model (see figure S4).

Sexual reproduction : It is possible to introduce other types of reproduction in the model presented in the main text. For instance, sexual reproduction would consist in picking randomly two parents and combining their α to produce an offspring. One intuitive way to do it is to have the offspring's α be an average of the parents'. Running simulations with sexual reproduction implemented produces the same set of mortality patterns than the one described in the main text (see figure S5). One could note as well that the distribution is more centered around the mode as the tail decreases towards $\alpha = 1$.

Maturation time : Maturation time can be introduced in the model by preventing reproduction to occur before a predetermined time t_r. Setting a such a window during which reproduction can occur, disfavors the high α. Indeed, the first reproductive events are mainly accomplished by α close to 1. As a result, the distributions after evolution show that the region close to 1 is more depleted in individuals. Yet, the corresponding mortality patterns are qualitatively similar to those observed with the other versions of the model (figure S6): the results are once again robust to such modifications of the model.

Heterogeneity arising during development : Implementation of maturation does not exactly modifies the initial adult heterogeneity. If one wants to account for specific developmentally induced heterogeneity, the deterministic model can be modified as follows. Keeping the notations from the main text, the stationary distribution of α, D_α, is now implicitly defined by the relation : $D_\alpha = T(C(D_\alpha))$ where T is the operator defined

in the main text and C is the operator describing the changes occurring during development in terms of heterogeneity. One interpretation of C could be for instance that low α fail to develop properly (because the process is too slow for instance) and therefore, C would enhance the frequency of high α in the population over the developmental period. Describing what could be a potential C is beyond the scope of the present paper. Yet, the mathematical framework is ready to be studied in the light of experimental data.

Maintaining heterogeneity

Maintaining population diversity

In this model, two phenomena contribute to maintain population heterogeneity: mutations and time-dependent competition.

A non zero mutation rate is expected to maintain a certain minimal diversity in the population. Yet, the expected distribution would show a peak at the optimal strategy (optimal α here) and be uniformly distributed elsewhere as mutations do not favor specific strategies. In the absence of mutations, population heterogeneity quickly moves from the initial uniform distribution to peak around one value of α (HRM). The key observation here is that the depletion occurring for high α is slower than the depletion for small α. In only three generations, there is no α left close to zero while a significant fraction remains close to one. This asymmetry can be explained by the reproduction process: high α always have a chance to reproduce at early ages because they are most likely to be alive, even if for a short period of time. During this small time-window, there is a chance that they would reproduce which is significantly higher than for small α. Second, the depletion around $\alpha = 1$ gets slower with generations. One way to represent the effect of mutations in figure S7 is to shift the whole distribution up and make it more flat. Therefore, at some point this shift and flattening exactly compensates the depletion in high α: the distribution stabilizes before reaching a single peak and presents an asymmetry between low α and high α.

The same phenomenon also occurs in the case of heterogeneity in aging timing but is no longer prevalent. As shown in figure S8, under low mutation rates, the distribution of α continues being bimodal in the HTM. Heterogeneity is maintained here because of

the time-dependent competition. The key idea is that the competition for reproduction depends on the individual alive at a given time. From the point of view of an individual with a small α value, competition is really strong at early ages because it competes with high α individuals. As time goes on, these individuals die out and the probability to reproduce increases for this individual. In sum, the optimal strategy changes with time.

Mathematical toy model Here, we derive a toy model to illustrate how traits in competition can be simultaneously maintained in a deterministic model. Let us consider a population of infinite size in which l traits are coexisting initially in equal proportions. Each trait i is characterized by a reproductive success a_i and a mortality function, which depends on its age. We are interested in the evolution of the vector $X(n) = ()$ where $x_i(n)$ is the proportion of individuals with trait i in the population at the generation n. To illustrate our point, we consider a discrete time here which corresponds to discrete instants for reproduction of the alive individuals.

Single time point for reproduction: In the case of a single reproduction opportunity, there is no aging occurring. Therefore, we can write:

$$x_i(n+1) = \frac{a_i x_i(n)}{\sum_{j=1}^{k} a_j x_j(n)}$$

. In this set-up, with X being the vector containing all x_i, we have

$$\lim_{n_g \to +\infty} X(n_g) = (0, \ldots, 0, 1, 0, \ldots, 0)$$

The 1 is simply located at the position corresponding to the highest a_i: in the absence of damage accumulation, higher investment in reproduction is the single strategy which invades the population. In a word, there is no coexistence possible in this competitive set-up.

Two time points for reproduction: Let us consider now two distinct times to reproduce. In between these two time points, individuals investing heavily in reproduction would die more than individuals investing heavily in maintenance, thus changing the competition set-up at the second time-point. For the sake of simplicity, let us consider two traits in competition. Each trait i is described by a parameter a_i and two parameters s_i^1 and s_i^2 which describe the survival at the two time points considered. Individuals with the

first trait invest in reproduction, so that $a_1 = 1$, but die off quickly (s_1^1=1 and s_1^2=0). Individuals with the second trait favor more maintenance, and therefore $a_2 < a_1$, s_2^1=1 and s_2^2>0. The evolution of population heterogeneity is described by:

$$\begin{cases} x_1(n+1) = \dfrac{\mu\, x_1(n)}{x_1(n) + a_2\, x_2(n)} + 0 \\ x_2(n+1) = \dfrac{\mu\, a_2\, x_2(n)}{x_1(n) + a_2\, x_2(n)} + (1 - s_2^2) \end{cases} \tag{1}$$

This set of differential equations depict a competition between traits 1 and 2 yet exhibit an equilibrium point at which both traits coexist. Indeed, the possible equilibria of this system are:

$$\begin{cases} x_1^* = 0 \quad \& \quad x_1^* = \ 1 - \dfrac{1 - s_2^2}{1 - a_2} \\ x_2^* = 1 \quad \& \quad x_2^* = \ \dfrac{1 - s_2^2}{1 - a_2} \end{cases} \tag{2}$$

In sum, two features contribute to maintain population heterogeneity in or models. First, we show that heterogeneity can be maintained because of stochastic processes in a non-symmetric manner. Second, we find that time-dependent competition can lead to a stable coexistence of multiple competing traits.

The Jacobian matrix of the system for the non-trivial equilibrium point is:

$$\frac{\mu a_2}{S_{a_2}^2} \begin{bmatrix} x_2^* & -\, x_1^* \\ -x_2^* & x_1 \end{bmatrix}$$

where $S_{a_2} = x_1^* + a_2\, x_2^* = (s_2^2)^2$. Therefore, the non-zero eigenvalue of this matrix is $\frac{a_2}{s_2^2}$. This ensures that this equilibrium is stable.

Three times competition: Here, we have three traits in competition and three times to compete. We denote a_i the reproductive success of each trait during the first opportunity to reproduce, and b_i during the second. Once again, we assume that trait 1 favors reproduction more than trait 2, which itself favours reproduction more than trait 3, the opposite being true for survival. With these notations, the system can be rewritten as:

$$\begin{cases} x_1(n+1) = & \dfrac{s_1^1\, x_1(n)}{x_1(n) + a_2\, x_2(n) + a_3\, x_3(n)} \\ x_2(n+1) = & \dfrac{s_2^1\, a_2\, x_2(n)}{x_1 + a_2\, x_2 + a_3\, x_3} + \dfrac{s_2^2\, b_2\, x_2(n)}{b_2\, x_2(n) + b_3\, x_3(n)} \\ x_3(n+1) = & \dfrac{s_3^1\, a_3\, x_3(n)}{x_1 + a_2\, x_2 + a_3\, x_3} + \dfrac{s_3^2\, b_3\, x_3(n)}{b_2\, x_2(n) + b_3\, x_3(n)} + s_3^3 \end{cases} \tag{3}$$

For the sake of simplicity, let us assume that $s_2^2 = s_3^2$. The equilibrium corresponding to $x_1^* \neq 0$, $x_2^* \neq 0$ and $x_3^* \neq 0$ is:

$$\begin{cases} x_1^* = & 1 - x_2^* - x_3^* \\ x_2^* = & \dfrac{s_2^2}{1 - a_2} - \dfrac{b_3}{b_2} x_3^* \\ x_3^* = & \dfrac{s_3^3}{1 - a_3 - \frac{b_3}{b2}(1 - a_2)} \end{cases} \tag{4}$$

Of course, x_1^*, x_2^* and x_3^* have to be between 0 and 1, which leads to the following constraints (with $\beta = \frac{b_3}{b2}$, $\bar{a}_2 = 1 - a_2$, $\bar{a}_3 = 1 - a_3$ and $c = \bar{a}_3 - \beta\bar{a}_2$):

$$\begin{cases} c > s_3^3 \\ 0 < s_2^2 c - \beta\, \bar{a}_2\, s_3^3 < \bar{a}_2\, c \\ 0 < s_2^2 c + (1 - \beta)\, \bar{a}_2\, s_3^3 < \bar{a}_2\, c \end{cases} \tag{5}$$

If we set $s_i^j = 1/3$ and $a_2 = 1/2$, then the above conditions reduce to:

$$\begin{cases} c > \beta\, \bar{a}_2 \\ c > 1/3 \\ c > 1 - \beta \\ c < 1 - \beta\, \bar{a}_2 \end{cases} \tag{6}$$

All the conditions of the system can be satisfied by a set of values for the couple (c, β) which corresponds to the grey area in figure S9. The coexistence of all three traits in the population is therefore possible.
